# Convergent reductions in interhemispheric functional, structural and callosal connectivity in Parkinson’s disease

**DOI:** 10.3389/fnagi.2025.1512130

**Published:** 2025-02-13

**Authors:** Erlei Wang, Yujing Jia, Luqi Cheng, Chengjie Mao, Yiqing Bao, Junkang Shen, Yuanchao Zhang, Guohua Fan

**Affiliations:** ^1^Department of Radiology, The Second Affiliated Hospital of Soochow University, Suzhou, China; ^2^School of Life and Environmental Sciences, Guilin University of Electronic Technology, Guilin, China; ^3^Department of Neurology, The Second Affiliated Hospital of Soochow University, Suzhou, China; ^4^School of Life Science and Technology, University of Electronic Science and Technology of China, Chengdu, China; ^5^College of Health Solutions, Arizona State University, Tempe, AZ, United States

**Keywords:** Parkinson’s disease, interhemispheric connectivity, lateralization, voxel mirrored homotopic connectivity, corpus callosum

## Abstract

**Background:**

Abnormal interhemispheric functional connectivity is frequently reported in Parkinson’s disease (PD), but its structural basis remains unclear. This study aimed to investigate changes in interhemispheric functional, structural, and callosal connectivity, as well as their interrelationships, in PD patients.

**Methods:**

The study included 57 PD patients and 50 healthy controls (HCs). Interhemispheric functional connectivity was evaluated using voxel mirrored homotopic connectivity (VMHC) derived from resting-state functional MRI, while structural connectivity was measured through homotopic cortical thickness covariance from T1-weighted MRI. The corpus callosum (CC), connecting bilateral regions with VMHC differences, was assessed using fractional anisotropy (FA) from diffusion MRI. Pearson’s correlation was used to evaluate the interrelationships among imaging data and their clinical relevance.

**Results:**

Compared to HCs, PD patients showed reduced VMHC and interhemispheric structural connectivity in similar brain regions, displaying a positive correlation trend between these measures. The affected regions encompassed the bilateral sensorimotor cortices (precentral gyrus, postcentral gyrus, and paracentral lobule) and posterior cortical areas, including the superior parietal lobule, supramarginal gyrus, precuneus, middle occipital gyrus, fusiform gyrus, as well as the superior and middle temporal gyri. FA in the CC, connecting regions with reduced VMHC, was also lower in PD patients. Additionally, interhemispheric structural, functional, and callosal connectivity reductions were, respectively, related to cognitive impairment, motor dysfunctions, and disease duration in PD.

**Conclusion:**

The study identified convergent reductions in interhemispheric functional, structural and callosal connectivity in PD patients, emphasizing the strong link between structural and functional brain abnormalities. Our findings may provide new insights into the pathophysiology of PD.

## Introduction

Parkinson’s disease (PD) is a prevalent neurodegenerative disorder, initially presenting with motor impairments on one side of the body, including tremor, rigidity, and bradykinesia (Cubo et al., 2020; Jeong et al., 2022). PD patients may also experience lateralized non-motor symptoms, such as pain, visuospatial deficits, and language impairments (Steinbach et al., 2023). Asymmetry in these symptoms is associated with uneven pathological changes between the hemispheres, including nigrostriatal dopaminergic degeneration and *α*-synuclein accumulation (Riederer and Sian-Hulsmann, 2012; Riederer et al., 2018). Increasing evidence indicates that abnormal interhemispheric interactions resulting from asymmetric pathology significantly impact PD symptoms (Spagnolo et al., 2013; Miller-Patterson et al., 2018; Wu et al., 2020).

Multimodal MRI techniques enable the *in vivo* assessment of interhemispheric connectivity. For instance, Voxel mirrored homotopic connectivity (VMHC) from resting-state functional MRI quantifies functional connectivity between each voxel in one hemisphere and its mirrored counterpart (Zuo et al., 2010). Diffusion MRI assesses the microstructural integrity of the corpus callosum (CC), the main white matter commissure linking the hemispheres (Yuan et al., 2020). Structural connectivity can also be inferred from the covariance of cortical morphology in homotopic regions, reflecting synchronized plasticity, shared pathological changes and genetic influences (He et al., 2007; Wang et al., 2021a). In PD patients, previous VMHC studies have identified specific patterns of VMHC reductions linked to various motor and non-motor symptoms, including different motor subtypes, impulse control disorders, depression, and apathy, underscoring its clinical relevance in PD (Hu et al., 2015; Luo et al., 2015; Wu et al., 2020; Gan et al., 2021; Zhang et al., 2022). Additionally, reductions in the microstructural integrity, thickness, or volume of the CC have been reported in PD patients with postural instability and gait disorder, cognitive impairment, and hallucinations (Wei et al., 2020; Amandola et al., 2022). Some studies have explored the relationship between VMHC changes and cortical thickness or gray matter asymmetry but found no evidence (Gan et al., 2021; Zhang et al., 2022). In contrast, structural connectivity inferred from morphological covariance offers a more direct perspective. No previous studies have specifically examined changes in structural interhemispheric connectivity in PD, and it remains unclear whether changes in structural, functional, and callosal connectivity are related or independent. Simultaneous investigation of these multimodal changes may offer new insights into the structural and functional interplay in PD.

This study aimed to investigate changes in interhemispheric functional, structural and callosal connectivity in PD patients compared to healthy controls (HCs). First, VMHC was assessed between the two groups to identify alterations in interhemispheric functional connectivity in PD patients. Second, differences in interhemispheric structural connectivity were assessed by analyzing deviations in the cortical thickness covariance of homotopic vertices in PD patients relative to normative values from the HC group. Third, callosal connectivity differences were examined by comparing the fractional anisotropy (FA) of the CC connecting regions with reduced VMHC. We hypothesized that PD patients would show similar reductions in both structural and functional interhemispheric connectivity and that the CC segments linking these regions would exhibit decreased FA in this population.

## Methods

### Participants

The study included 107 participants, comprising 57 individuals with PD and 50 HCs, who were demographically matched. PD patients were diagnosed by an experienced specialist at the Second Affiliated Hospital of Soochow University, following the criteria of the United Kingdom Parkinson’s Disease Society Brain Bank (Hughes et al., 1992). Exclusion criteria included serious psychiatric or neurological disorders other than PD, cardiovascular or metabolic diseases, and cognitive impairment. PD symptoms, disease progression, and cognitive abilities were systematically assessed using the Unified Parkinson’s Disease Rating Scale motor section (UPDRS-III) (Fahn and Elton, 1987), Hoehn and Yahr staging (Hoehn and Yahr, 1967), and the MMSE (Folstein et al., 1975). Meanwhile, HCs without a family history of PD, or a history of neurological or psychiatric disorders were recruited through advertisements. The study received approval from the Medical Ethics Committee of the Second Affiliated Hospital of Soochow University. All participants provided written informed consent prior to participation.

### MRI acquisition

Imaging was performed using a 3 T Philips Achieva scanner at Philips, Best, The Netherlands, equipped with a 32-channel head coil. To minimize head motion and dampen scanner noise, foam pads and earplugs were utilized. The MRI acquisition protocols included the following specifics: For T1-weighted MRI imaging, a fast field echo sequence was employed, capturing 155 sagittal slices with a repetition time (TR) of 7.1 ms, echo time (TE) of 3.5 ms, an 8° flip angle, and a field of view (FOV) of 220 × 220 mm^2^. The acquisition matrix was set to 220 × 199, with a reconstructed matrix of 352 × 352, and voxel dimensions of 0.63 × 0.63 × 1 mm^3^. Diffusion MRI incorporated one non-diffusion-weighted image (b = 0 s/mm^2^) and 16 diffusion-weighted images (b = 1,000 s/mm^2^) using a single-shot echo-planar imaging sequence, specified with a TR of 6,000 ms, TE of 120 ms, a 90° flip angle, FOV of 220 × 220 mm^2^, matrix size of 128 × 128, and voxel dimensions of 1.72 × 1.72 × 3 mm^3^. Additionally, resting-state functional MRI data were captured over 36 axial slices with TR/TE parameters of 2000/30 ms, a 90° flip angle, matrix size of 64 × 64, FOV of 220 × 220 mm^2^, and voxel dimensions of 3.4 × 3.4 × 4 mm^3^, totaling 200 volumes.

To assess callosal connectivity associated with VMHC differences using probabilistic tractography, an additional cohort of 15 HCs aged 60–71 years underwent scanning with T1-weighted and diffusion MRI images. This secondary dataset was acquired using a 3 T Siemens Prisma scanner in Erlangen, Germany, equipped with a 64-channel head and neck coil. The T1-weighted imaging followed a magnetization-prepared rapid-acquisition gradient echo sequence, set with a TR of 2,300 ms, TE of 2.34 ms, an inversion time of 900 ms, an 8° flip angle, FOV of 256 × 256 mm^2^, matrix size of 256 × 256, and voxel dimensions of 1 × 1 × 1 mm^3^. DTI parameters included a half-coverage Cartesian q-space grid sampling method with a radial grid size of 4, collecting 128 diffusion-weighted images and 18 different b-values ranging from 0 to 3,000 s/mm^2^. Detailed DTI acquisition settings were TR of 3,900 ms, TE of 72 ms, a 90° flip angle, FOV of 220 × 220 mm^2^, and a matrix size of 110 × 110 with voxel dimensions of 2 × 2 × 2 mm^3^.

### VMHC analysis

Resting-state functional MRI data were preprocessed using the Data Processing & Analysis for Brain Imaging (DPABI) software^1^ (Yan and Zang, 2010), run in MATLAB R2018b (MathWorks). The preprocessing protocol included: (1) discarding the first 10 volumes to allow for signal equilibration; (2) correcting for differences in slice acquisition timing; (3) realigning volumes to compensate for participant head movements; (4) co-registering each participant’s anatomical T1 images with their functional images; (5) segmenting the co-registered T1 images into gray and white matter and normalizing to the Montreal Neurological Institute (MNI) reference space; (6) applying the same spatial normalization parameters to the functional images; (7) smoothing the images using a Gaussian filter with a 6 mm full width at half maximum to increase signal-to-noise ratio; (8) applying a band-pass filter (0.01–0.1 Hz) to reduce the effect of low-frequency drifts and high-frequency physiological noise; and (9) removing confounding factors such as fluctuations related to white matter and cerebrospinal fluid, along with six parameters of head motion. Data from participants exhibiting more than 2 mm of translational or 2° of rotational head movement were excluded from further analysis. Additionally, the mean framewise displacement (FD) was calculated for each subject and used as a covariate in subsequent statistical analysis.

For VMHC analysis, a specialized symmetric T1 template was constructed. This involved averaging the spatially normalized T1 images across all participants to form a mean T1 template. This mean template was then mirrored along the left–right axis and averaged again with its original to produce a symmetric T1 template tailored for this group. Each participant’s preprocessed functional MRI data was then aligned to this symmetrical template (Zuo et al., 2010). For each symmetrical voxel pair across hemispheres, Pearson’s correlation coefficients were calculated to quantify homotopic connectivity. These coefficients were subsequently transformed into z-scores using the Fisher Z transformation to facilitate more robust statistical comparison across subjects.

### Homotopic structural connectivity analysis

T1-weighted MRI data preprocessing was executed using the Computational Anatomy Toolbox 12^2^ (Li et al., 2020; Wang et al., 2021a; Wang et al., 2021b). Initially, all DICOM images were converted to NIfTI format and carefully inspected for motion artifacts or other discrepancies. The preprocessing steps incorporated bias-field correction to address inhomogeneities; segmentation of brain tissues into gray matter, white matter, and cerebrospinal fluid; followed by normalization using the Diffeomorphic Anatomical Registration Through Exponentiated Lie algebra algorithm to ensure accurate overlay with standard templates. Cortical thickness was quantified using a projection-based thickness analysis, after which cortical thickness maps were adapted to the 32 k Human Connectome Project standard mesh, ensuring accurate bilateral alignment for direct hemispheric comparison (Dahnke et al., 2013). These maps were then smoothed with a 15 mm full-width-at-half-maximum (FWHM) Gaussian kernel to prepare for further analyses.

The analysis of interhemispheric structural connectivity differences began by establishing a baseline structural connectivity map (PCCn) for the HC group. This was achieved by calculating Pearson’s correlation coefficients for cortical thickness across corresponding hemispheric regions (homotopic vertices). To assess the impact of disease on connectivity, the analysis was extended by incorporating a patient’s data into the HC group and recalculating the correlation coefficients, thus generating a modified connectivity map (PCC_n + 1_). The difference in connectivity, denoted as ΔPCCn (PCC_n + 1_ - PCCn), was then calculated. This difference typically exhibits a ‘volcano distribution’, characterized by tails that resemble those of a normal distribution. The statistical significance of changes in connectivity was determined using a Z-score formula: $z=\frac{\Delta PCC_{n}}{\left(1-\Delta PCC_{n}^{2}\right)/\left(n-1\right)}$, where ΔPCCn is the deviation from the normative pattern, and n is the number of controls. Z-score maps were then tested against a null hypothesis of zero difference, with significant negative values indicating reduced structural connectivity between hemispheres in PD patients. Before these steps, potential confounding variables such as age and gender were statistically removed from the cortical thickness data to avoid biased results (Liu et al., 2016).

### Callosal connectivity analysis

Diffusion MRI data were processed using the Functional MRI of the Brain’s Software Library.^3^ Initially, corrections were applied for head movements and distortions caused by eddy currents using FSL’s eddy correction tool. Non-cerebral elements were then removed from these corrected images employing FSL’s brain extraction tool. Following this, a fractional anisotropy (FA) map of each voxel was constructed by applying a tensor model with the DTIFIT function within FSL. The final step involved aligning the FA maps to the MNI standard space through a nonlinear registration process to ensure accurate anatomical alignment.

For the additional dataset, Diffusion MRI preprocessing mirrored the procedures outlined previously. Once preprocessing was complete, voxel-level fiber orientation distributions were calculated utilizing FSL’s Bedpostx utility. This data facilitated probabilistic tractography aimed at delineating the CC segments linking areas with notable VMHC disparities. Each hemisphere’s regions exhibiting significant VMHC differences were isolated to create distinct seed masks, initiating fiber tracking across the whole brain from these locations to generate 5,000 streamline samples per voxel (Wang et al., 2017). Voxels containing at least one streamline were preserved. The tracts were transformed into MNI space and averaged across participants to generate a population-based probability map of WM tracts. A threshold of *p* > 20% was applied to binarize the population-based probability map for each hemisphere separately. A union operation was applied to the tracts of the seed mask in each hemisphere, creating a binary image representing all possible tracts connected to the seed masks. An intersection operation with the CC mask from the JHU white matter atlas was then performed to identify the CC region connecting bilateral areas with VMHC differences. The mean FA of that CC region was extracted and compared between PD patients and matched HCs.

### Statistical analysis

Statistical evaluations were performed using SPSS version 22.0 (SPSS Inc., Chicago, IL, United States). Demographic and clinical characteristics between PD patients and HCs were compared using independent t-tests for continuous data and chi-square tests for categorical data. A significance level was maintained at *p* < 0.05 for all tests.

VMHC disparities between groups were analyzed using a two-sample t-test on a voxel-by-voxel basis within the DPABI platform, adjusting for age, gender, and mean FD as nuisance variables. These analyses adhered to stringent criteria, reporting significance at a voxel height of *p* < 0.001 and a cluster significance of *p* < 0.05, applying Gaussian random field theory for correction.

Changes in homotopic structural connectivity in PD patients were examined using a one-sample t-test within the SPM12. The derived statistical maps underwent correction for multiple comparisons using a threshold-free cluster enhancement technique with 5,000 permutations, setting family-wise error correction at *p* < 0.05.

Differences in mean FA within the CC segments linked with VMHC variations were quantitatively assessed using a two-sample t-test to contrast PD patients with HCs, with thresholds for significance also set at *p* < 0.05.

Additionally, Pearson’s correlation was used to evaluate the interrelationships among imaging data and their clinical relevance, such as disease duration, UPDRS-III scores, and MMSE scores. These correlations were considered statistically significant at *p* < 0.05.

## Results

### Demographic and clinical data

Details regarding demographic and clinical characteristics are shown in Table 1. There were no significant differences in age, gender, or education level between the two groups (*p* = 0.142, *p* = 0.373, and *p* = 0.328, respectively). However, the average MMSE scores were significantly lower in the PD group compared to the HCs (*p* < 0.001).

**Table 1:** Demographic and clinical data of the participants.

	PD (*n* = 57)	HCs (*n* = 50)	*P*
Age (years)	61.68 ± 7.04	63.56 ± 5.92	0.142
Gender (male/female)	(30/27)	(22/28)	0.373
Education (years)	7.72 ± 3.78	8.46 ± 4.03	0.328
Disease duration (years)	3.25 ± 2.12		
UPDRS III score	22.42 ± 12.79		
H&Y	1.88 ± 0.62		
MMSE score	27.67 ± 1.37	28.92 ± 1.14	0.001
LEED (mg)	392.97 ± 140.65		

All continuous variables are given as mean (standard deviation). PD, Parkinson’s disease; HCs, healthy controls; UPDRS-III, Unified Parkinson’s Disease Rating Scale (motor section); H&Y, Hoehn and Yahr staging; MMSE, Mini-Mental State Examination; LEED, levodopa equivalent daily dose.

### Decreased VMHC in PD

**Figure 1:**
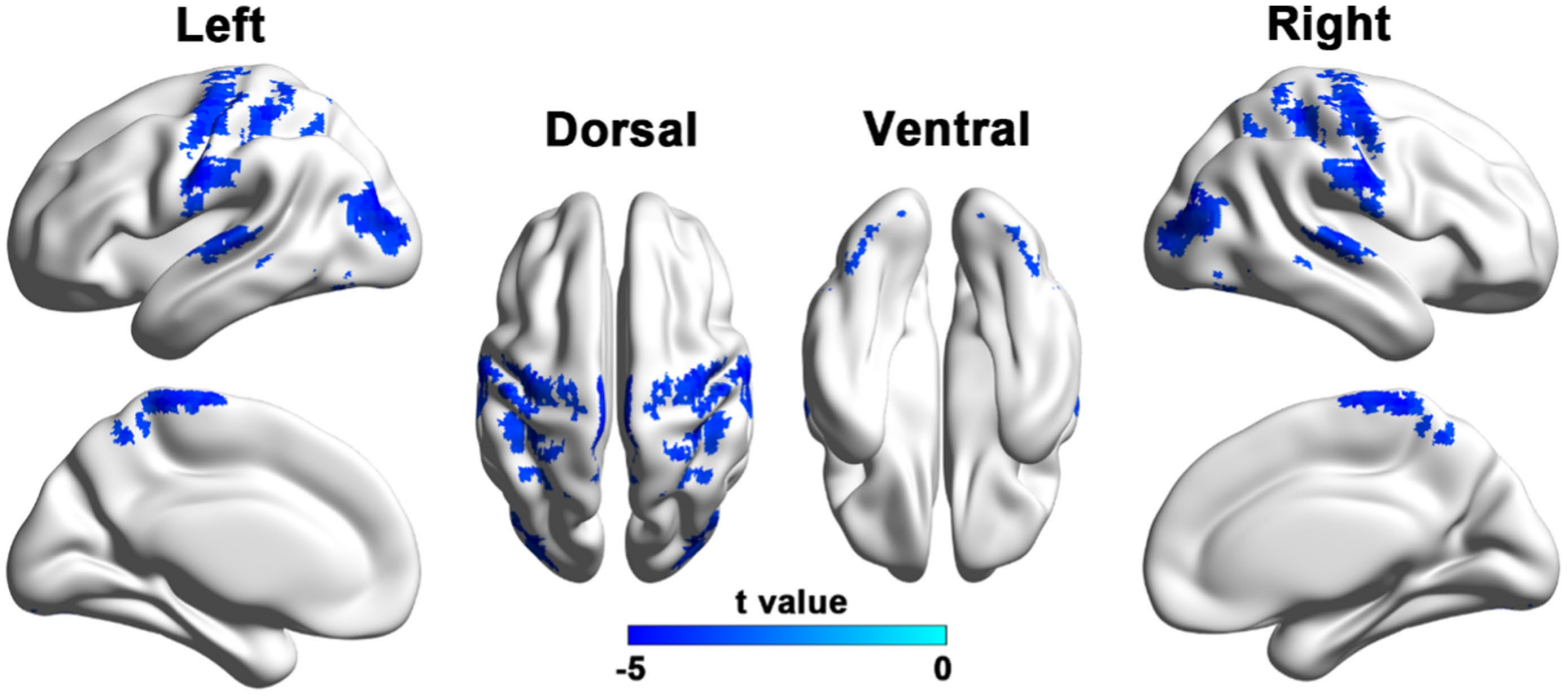
Brain regions with reduced VMHC in PD patients compared to HCs. Results were reported with a height threshold of *p* < 0.001 and a cluster threshold of *p* < 0.05, using GRF correction. The color bar shows the T values for between-group contrasts.

PD patients exhibited reduced VMHC in the bilateral sensorimotor regions, including the precentral and postcentral gyri, as well as the paracentral lobule. Additionally, reductions were observed in the posterior cortical areas, such as the superior parietal lobule, precuneus, supramarginal gyrus, middle occipital gyrus, and the superior and middle temporal gyri, as shown in Figure 1 and Table 2.

**Table 2:** Brain regions showing decreased VMHC in patients with PD relative to HCs.

Regions	MNI Coordinates			Peak t-score	No. Voxels
	x	y	z		
PostCG/PreCG/SPG/PreCUN/SupraMG/ParaCG	±60	−12	30	−5.3079	768
STG/MTG	±57	−24	6	−4.5767	103
MOG/MTG	±39	−84	21	−5.1902	191
MOG	±30	−75	−24	−4.2651	75

PostCG, postcentral gyrus; PreCG, precentral gyrus; SPG, superior parietal gyrus; PreCUN, precuneus; SupraMG, supramarginal gyrus; ParaCG, paracentral_lobule; STG, superior temporal gyrus; MTG, middle temporal gyrus; MOG, middle occipital gyrus.

### Decreased homotopic structural connectivity in PD

**Figure 2:**
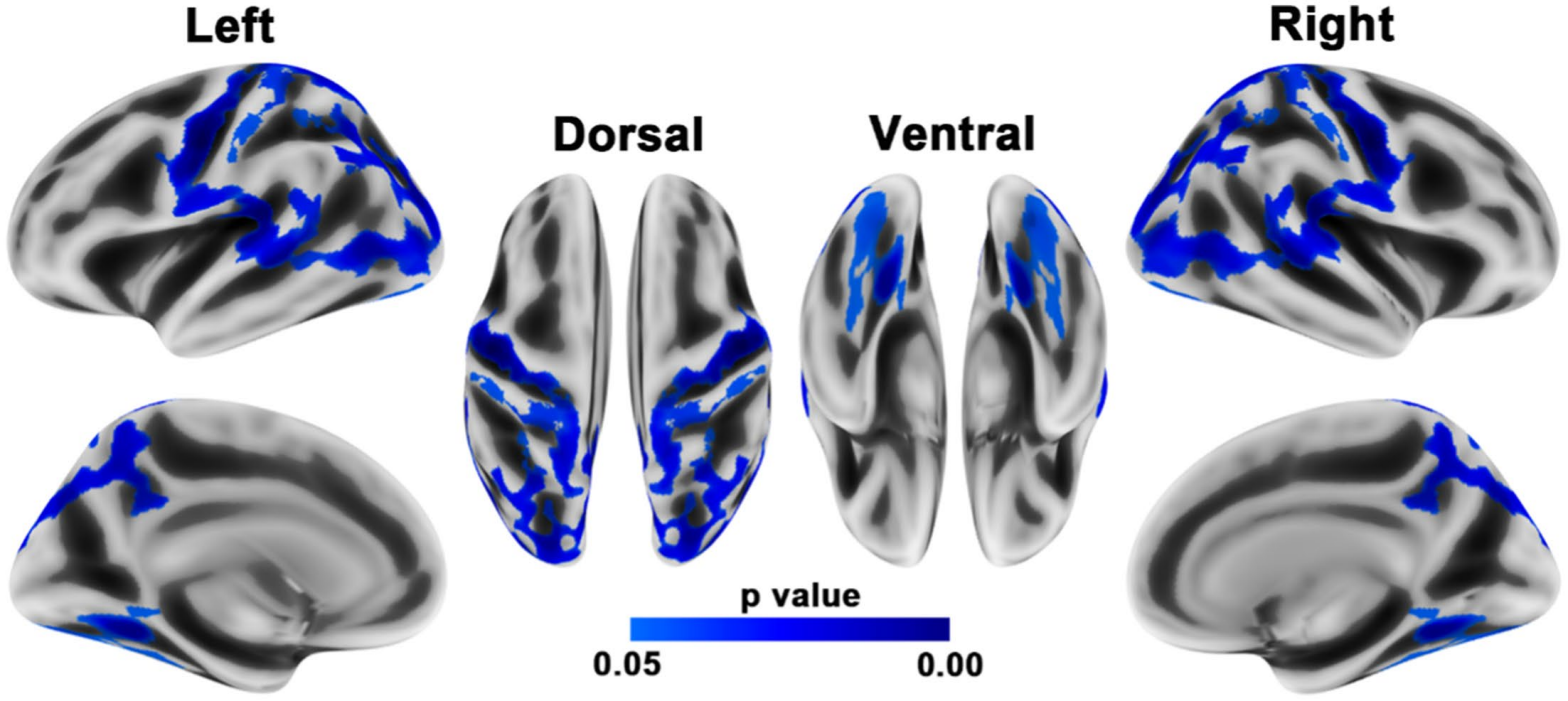
Brain regions with reduced homotopic structural connectivity in PD patients compared to HCs. The results were adjusted for multiple comparisons using TFCE. Statistical significance was set at p < 0.05 with family-wise error correction. The color bar displays the TFCE-corrected *p*-values.

When compared to HCs, PD patients showed reduced homotopic structural connections within both the sensorimotor regions (precentral and postcentral gyri) and posterior brain regions, such as the superior and inferior parietal lobules, precuneus, supramarginal gyrus, middle occipital gyrus, and superior temporal gyrus (Figure 2; Table 3).

**Table 3:** Brain regions showing decreased homotopic structural connectivity in patients with PD compared with HCs.

*p*- value	Size (vertices)	Overlap with regions of the DK atlas	Regions
0.00361	5,040	18%	SPG
		15%	PostCG
		15%	PreCG
		10%	MOG
		10%	IPG
		8%	PreCUN
		7%	STG
		6%	SupraMG
		5%	bSTS
		2%	MTG
		1%	IC
0.01004	608	47%	FG
		35%	LG
		15%	MOG
		3%	ParaHIPP

SPG, superior parietal gyrus; PreCG, precentral gyrus; PostCG, postcentral gyrus; MOG, middle occipital gyrus; IPG, inferior parietal gyrus; PreCUN, precuneus; STG, superior temporal gyrus; SupraMG, supramarginal gyrus; bSTS, banks of the superior temporal sulcus; MTG, middle temporal gyrus; IC, isthmus cingulate; FG, fusiform gyrus; LG, lingual gyrus; ParaHIPP, parahippocampal gyrus.

### Decreased callosal connectivity in PD

**Figure 3:**
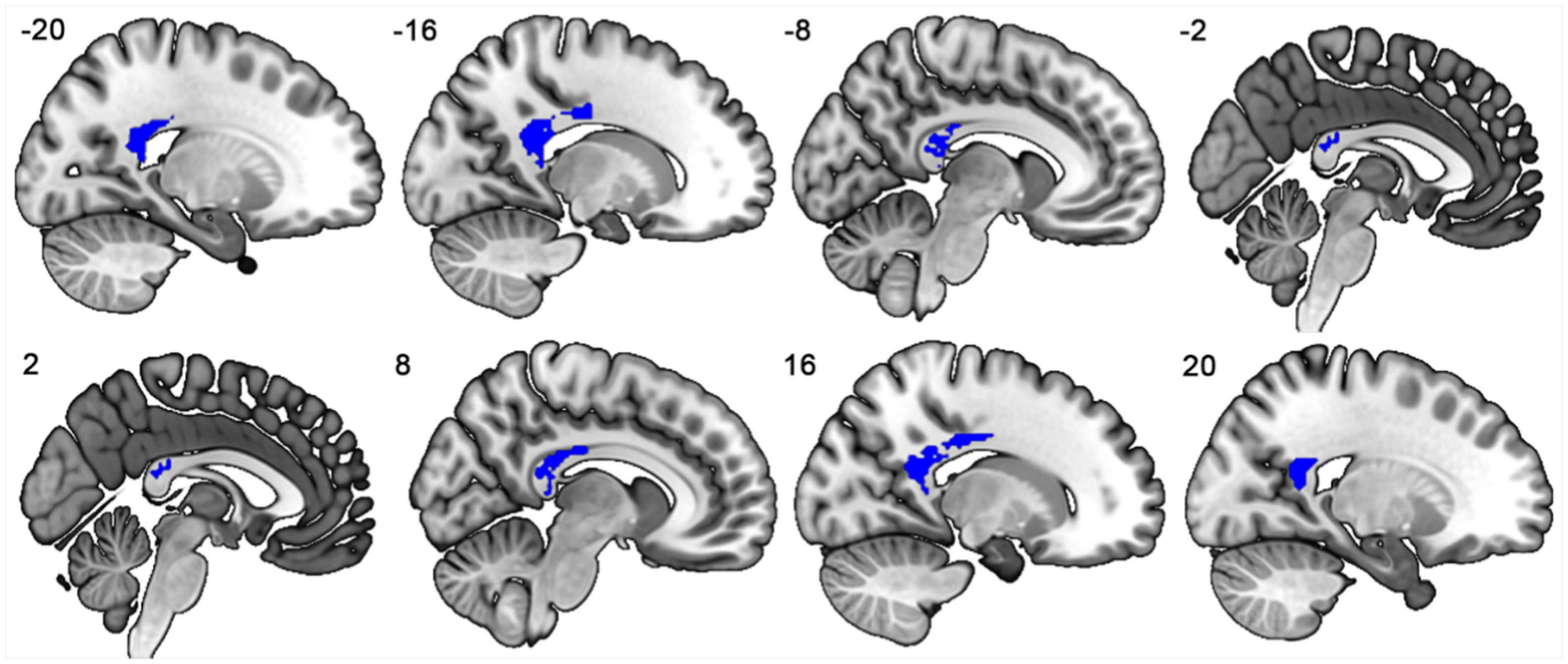
The mid-posterior and posterior subregions of the corpus callosum connecting the bilateral regions showing significant VMHC reductions.

**Figure 4:**
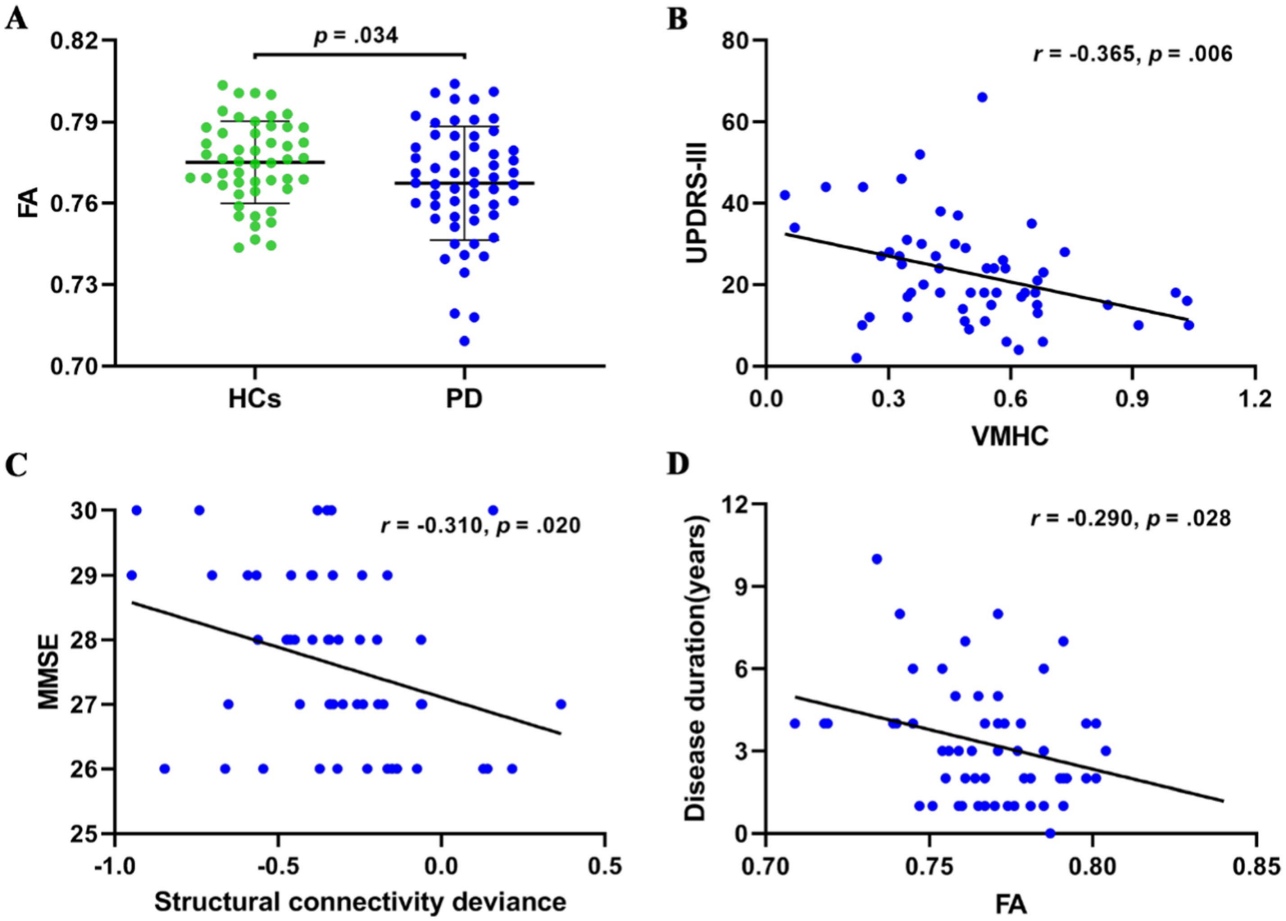
**(A)** Error bar plots showing FA in the mid-posterior and posterior subregions of the corpus callosum for PD patients and HCs. **(B)** Negative correlation between VMHC and UPDRS-III scores in PD patients. **(C)** Negative correlation between structural connectivity deviation and MMSE scores in PD patients. **(D)** Negative correlation between FA in the mid-posterior and posterior subregions of the corpus callosum and disease duration in PD patients. PD, Parkinson’s disease; HCs, healthy controls; FA, fractional anisotropy; UPDRS-III, The Unified Parkinson’s Disease Rating Scale motor section; VMHC, voxel mirrored homotopic connectivity; MMSE, Mini-Mental State Examination scores.

When compared to HCs, PD patients showed FA reductions in the mid-posterior and posterior segments of the CC, which connect regions with VMHC reductions (Zheng et al., 2022), as shown in Figure 3 and Figure 4A.

### Correlation analysis

In the PD group, a trend toward a positive correlation was observed between reductions in VMHC and homotopic structural connectivity (*r* = 0.251, *p* = 0.062). Significant negative correlations were found between VMHC values and UPDRS-III scores (*r* = −0.365, *p* = 0.006), between deviations in structural connectivity and MMSE scores (*r* = −0.310, *p* = 0.020), and FA in the mid-posterior and posterior CC and disease duration (*r* = −0.290, *p* = 0.028), as shown in Figure 4.

## Discussion

This study used multimodal MRI to compare interhemispheric functional, structural, and callosal connectivity between PD patients and HCs. The key findings are as follows. First, PD patients exhibited similar reductions in VMHC and homotopic structural connectivity compared to HCs, with a trend toward a positive correlation between these reductions. Second, PD patients had lower FA in the mid-posterior and posterior CC connecting the bilateral regions with VMHC reductions. Third, reduced interhemispheric functional, structural, and callosal connectivity were linked to motor dysfunction, cognitive decline, and disease duration in PD patients. These findings provide new insights into the strong relationship between structural and functional brain abnormalities in PD.

PD patients showed significantly reduced VMHC and homotopic structural connectivity in the precentral and postcentral gyri compared to HCs. This aligns with previous findings of decreased VMHC in similar cortical regions in PD (Hu et al., 2015; Luo et al., 2015; Wu et al., 2020; Gan et al., 2021), and is supported by reports of gray matter atrophy and functional hypoactivity in these regions (Burciu et al., 2015; Li et al., 2020). The precentral gyrus, the primary motor cortex, controls voluntary motor movement on the contralateral side. Coordinated movements require balanced inhibitory and excitatory interactions between the bilateral primary motor cortices (Carson, 2005; Beaule et al., 2012). However, this balance may be disrupted in unilateral neurological conditions like amyotrophic lateral sclerosis, stroke, and PD (Karandreas et al., 2007; Gerges et al., 2022; Steinbach et al., 2023), manifesting as reduced interhemispheric connectivity. Furthermore, the unaffected hemisphere may exert heightened inhibitory influence over the affected hemisphere, further exacerbating this imbalance and potentially increasing the lateralization of motor impairments in PD (e.g., tremors, bradykinesia, and rigidity). Additionally, motor deficits, including mirror movements and impaired bilateral motor coordination, may also result from the reduced interhemispheric connectivity in PD (Spagnolo et al., 2013). Furthermore, the postcentral gyrus, as the primary somatosensory cortex, processes sensory input from the contralateral side and integrates inputs for fine motor control. Interhemispheric interactions between the bilateral primary somatosensory cortices are essential for integrating somatosensory inputs from both sides of the body, facilitating precise movement (Borich et al., 2015; Tame et al., 2016)^.^ Sensory disturbances, such as haptic and proprioceptive deficits, are common in PD, and impaired sensorimotor integration may exacerbate motor symptoms (Konczak et al., 2009; Conte et al., 2013). Our findings suggest that reduced homotopic connectivity in sensory and motor cortices reflects impaired interhemispheric interactions, likely driven by unbalanced inhibitory and excitatory signals, contributing to PD sensorimotor deficits. This is further supported by the significant positive correlation between VMHC and UPDRS-III motor scores.

PD patients exhibited lower VMHC and homotopic structural connectivity in posterior cortical regions, including the superior and inferior parietal lobules, precuneus, supramarginal gyrus, lateral occipital gyrus, and superior temporal gyrus, compared to HCs. This finding aligns with previous studies showing significant posterior cortical atrophy, hypometabolism, and hypoperfusion in PD patients, particularly those with cognitive impairment and visual dysfunction (Wallin et al., 2007; Hosokai et al., 2009; Garcia-Diaz et al., 2018). The posterior cortical regions are primarily involved in visuospatial and visuo-perceptual functions. Previous studies have found decreased interhemispheric functional connectivity between bilateral posterior cortical regions in conditions involving cognitive impairment and visual deficits, such as Alzheimer’s disease, early blindness, and unilateral acute open globe injury (Hou et al., 2017; Li et al., 2018; Luo et al., 2018; Ye et al., 2018). In PD patients, cognitive impairment and visual dysfunction are among the most frequently reported non-motor symptoms, with visual dysfunction being a significant predictor of dementia (Hamedani et al., 2020). Therefore, our findings of reduced VMHC and homotopic structural connectivity in posterior cortical regions may contribute to cognitive and visual impairments in PD patients by disrupting interhemispheric interactions, which may facilitate the lateralization and integration of these functions. The negative correlation between deviations in structural connectivity and MMSE scores further corroborates these findings.

Additionally, PD patients exhibited lower FA in the mid-posterior and posterior subregions of the CC that connect bilateral regions with VMHC reductions compared to HCs. This finding aligns with previous reports of reduced microstructural integrity, volume, and thickness of the CC in PD patients, with alterations in specific subsections associated with motor and non-motor symptoms such as postural instability, gait disorders, cognitive impairment, and hallucinations (Chan et al., 2014; Goldman et al., 2017; Bledsoe et al., 2018; Zarkali et al., 2020). Notably, our findings primarily involved the splenium of the CC, which connects the bilateral parietal, temporal, and occipital cortices and plays a crucial role in the interhemispheric integration of cognitive and visual information (Raybaud, 2010; Blaauw and Meiners, 2020). For instance, Goldman et al. found that reduced splenium volume in the corpus callosum was associated with memory and visuospatial impairments in PD patients (Goldman et al., 2017). Furthermore, macrostructural changes in the splenium have been observed in PD patients experiencing hallucinations compared to those without. Thus, reduced FA in this region may indicate cognitive and visual impairments in our PD cohort (Zarkali et al., 2020). Thus, reduced FA in this region may indicate cognitive and visual impairments in our PD cohort. Our study identified a significant negative correlation between FA in the mid-posterior and posterior regions of CC and disease duration in PD patients, aligning with two previous longitudinal studies in early PD (Taylor et al., 2018; Amandola et al., 2022). These findings suggest that degeneration of the callosal microstructure could serve as a potential biomarker for monitoring disease progression in PD.

Overall, this study identified convergent reductions in interhemispheric connectivity in PD patients compared to HCs using functional, structural, and diffusion MRI data. Our results build on previous studies demonstrating a close association between structural and functional brain abnormalities in PD patients. For instance, previous research has shown that structural covariance, functional networks, and white matter networks share similar topological properties and organizational principles in PD, accompanied by reduced global information integration (Baggio et al., 2014; Koirala et al., 2019; Wang et al., 2021a). Previous studies have also found that the distribution of cortical atrophy depends on functional and white matter connectivity to the subcortical disease reservoir in PD (Yau et al., 2018). However, the relationship between changes in brain structure and function may not be one-to-one. The absence of significant correlations between callosal FA and changes in homotopic structural and functional connectivity suggests that alternative commissural connections, such as the anterior commissures and subcortical structures, may compensate for communication between the two hemispheres (Beaule et al., 2015; Yuan et al., 2020). The neural mechanisms underlying decreased interhemispheric connectivity in PD are unclear and may involve three types of pathological changes. First, PD is characterized by asymmetric degeneration of dopaminergic neurons in the substantia nigra pars compacta and dopaminergic denervation in the cortex, leading to different reorganizations of cortical function and structure in the two hemispheres (Riederer and Sian-Hulsmann, 2012; Riederer et al., 2018). Second, the propagation of *α*-synuclein pathology differs between homotopic regions in the two hemispheres, resulting in uneven cortical atrophy and dysfunction (Borghammer, 2021). Third, direct callosal degeneration caused by α-synuclein aggregates or Wallerian degeneration may disrupt interhemispheric structural and functional connectivity in PD (O’Keeffe and Sullivan, 2018; Zhang et al., 2023). It is likely that a combination of subcortical, cortical, and transcallosal degeneration contributes to the reductions in interhemispheric connectivity in PD patients.

This study has several limitations. First, as a cross-sectional study, it does not reveal the dynamic profiles of interhemispheric connectivity changes in PD patients. Second, most PD patients in our study were on dopaminergic medications, and we cannot rule out the possibility that these medications influenced our results. Third, subgroup analysis was not possible due to the relatively small sample size. Fourth, due to the limited number of gradient directions in our DTI data, an additional dataset with a higher number of gradient directions was used for fiber tractography. Additionally, different multiple comparison methods were applied to the statistical analysis of functional and structural data, which could influence the results to some extent. Fifth, all interhemispheric connectivity analyses were based on unidirectional connectivity, preventing us from determining the excitatory or inhibitory nature of interhemispheric influences. Future analyses of interhemispheric effective connectivity using dynamic causal modeling are needed to address this issue further. Finally, we cannot determine whether changes in interhemispheric callosal connections are homotopic or heterotopic (Szczupak et al., 2023), despite conducting VMHC and structural connectivity analyses to infer homotopic connectivity between the hemispheres. Future studies are necessary to explore this issue further.

In conclusion, the study identified consistent reductions in interhemispheric functional, structural and callosal connectivity in PD patients, emphasizing the strong link between structural and functional brain abnormalities. Our findings may provide new insights into the pathophysiology of PD.

## Data Availability

The raw data supporting the conclusions of this article will be made available by the authors, without undue reservation.

## Footnotes

^1^ http://rfmri.org/dpabi

^2^CAT12, http://dbm.neuro.uni-jena.de/cat12/.

^3^FSL, accessible at https://fsl.fmrib.ox.ac.uk/fsl/.
